# New‐onset sarcoidosis in a patient with long COVID

**DOI:** 10.1002/ccr3.9186

**Published:** 2024-08-10

**Authors:** Guillermo Rodriguez‐Nava, Vanessa El Kamari, Harvey Chang, Goar Egoryan, Hector F. Bonilla

**Affiliations:** ^1^ Division of Infectious Diseases and Geographic Medicine, Department of Medicine Stanford University School of Medicine Stanford California USA; ^2^ Department of Pathology Memorial Medical Center Modesto California USA; ^3^ Division of Oncology, Department of Medicine Stanford University School of Medicine Stanford California USA; ^4^ Stanford Post‐Acute COVID‐19 Syndrome Clinic Stanford Health Care Stanford California USA; ^5^ Stanford Myalgic Encephalomyelitis/Chronic Fatigue Syndrome Clinic Stanford Health Care Stanford California USA

**Keywords:** autoimmune, COVID‐19, long COVID, sarcoidosis

## Abstract

Long COVID, often following SARS‐CoV‐2 infection, may stem from sustained inflammation, overlapping with autoimmune diseases like sarcoidosis. Though specific treatments lack, this link could shape future diagnostic and therapeutic methods.

## 1 INTRODUCTION

Post‐acute sequelae of SARS‐CoV‐2 infection (PASC), also known as long COVID, is a complex medical condition that persists in selected patients after a SARS‐CoV‐2 infection.^1^ Although the definition of PASC is still evolving, the Centers for Disease Control and Prevention (CDC) defines it as the presence of persistent symptoms beyond 28 days, while the World Health Organization and the UK Government's Office for National Statistics consider symptoms lasting for 12 weeks or longer.^2^, ^3^, ^4^ This condition is characterized by over 200 associated symptoms, including hair loss, fever, fatigue, post‐exertional malaise, cognitive difficulties, mood disorders, sleep disturbances, headaches, palpitations, chest pain, and shortness of breath.^1^, ^5^ PASC symptoms can vary from mild to severe, significantly impacting daily activities and work obligations. Severe symptoms usually peak within the first 28 days post‐infection, while mild to moderate symptoms can persist for weeks afterward.^6^

According to the National Center for Health Statistics, the estimated prevalence of long COVID in the United States is 15.7%, and it is more commonly observed in females. This is likely influenced by biological factors like the X chromosome and sex hormone differences, alongside a pronounced innate and adaptive immune response that moderates acute COVID‐19 in females but increases susceptibility to inflammatory and autoimmune diseases.^7^, ^8^ Long COVID also often coexists with conditions such as diabetes, obesity, and chronic pulmonary or renal disease.^7^ One prevailing hypothesis to explain long COVID is an ongoing and sustained inflammatory response.^9^, ^10^ Following SARS‐CoV‐2 infection, various rheumatological and autoimmune diseases have been reported. These manifestations are diverse, ranging from organ‐specific to systemic autoimmune and inflammatory responses.^11^, ^12^ Organ‐specific manifestations include cutaneous vasculitis, immune thrombocytopenic purpura, transverse myelitis, and Guillain–Barré syndrome.^11^, ^12^ On the other hand, systemic autoimmune and inflammatory conditions encompass systemic vasculitis, multisystem inflammatory syndrome, hemophagocytic lymphohistiocytosis, and systemic lupus erythematosus.^11^, ^12^ Furthermore, there have been reported cases of sarcoidosis associated with SARS‐CoV‐2 infection.^13^, ^14^, ^15^, ^16^, ^17^, ^18^, ^19^, ^20^, ^21^, ^22^, ^23^, ^24^

Sarcoidosis is a multi‐systemic inflammatory disorder characterized by the formation of non‐caseating granulomas, which can affect different organs, including the lungs, regional lymph nodes, skin, liver, central nervous system, and eyes. It can affect individuals of all ages and races.^25^ The underlying cause of sarcoidosis remains unknown, but it is believed that various factors, including infections, contribute to its development and pathogenesis.^26^

We report the case of a previously healthy White male who developed long COVID and mediastinal lymphadenopathy after acute SARS‐CoV‐2 infection. ndobronchial ultrasound‐guided transbronchial needle aspiration revealing non‐caseating granulomas and high plasma ACE levels consistent with sarcoidosis.

## 2 CASE HISTORY/EXAMINATION

On December 24, 2020, a previously healthy 35‐year‐old white male presented to the office with shortness of breath, body aches, cough, nasal congestion, and with a temperature of 37.5°C. On presentation, he was found to be vitally stable and saturating at 95% on ambient air. Laboratory analyses were unremarkable, and a chest x‐ray showed bilateral patchy airway opacities. A SARS‐CoV‐2 polymerase‐chain reaction (PCR) from a nasopharyngeal swab was positive, confirming the diagnosis of mild COVID‐19. The patient was discharged on albuterol nebulizer, prednisone 40 mg daily for 5 days, and levofloxacin 750 mg daily for 5 days.

Following his initial episode of COVID‐19, the patient continued to experience persistent fatigue. In February 2021, he experienced recurrent chest congestion, tightness of the lungs, a productive cough, and a fever of 38.7°C. A SARS‐CoV‐2 PCR from a nasopharyngeal swab resulted negative, and he was empirically treated for suspected atypical pneumonia with azithromycin 500 mg on the first day, followed by 250 mg for 4 days. Two months later, he experienced another episode of generalized body aches, night sweats, and fevers up to 39.2°C, without cough, shortness of breath, or chest pain, which persisted for 2 weeks. Another SARS‐CoV‐2 PCR from a nasopharyngeal swab was performed, resulting in a positive result for the second time.

**FIGURE 1:**
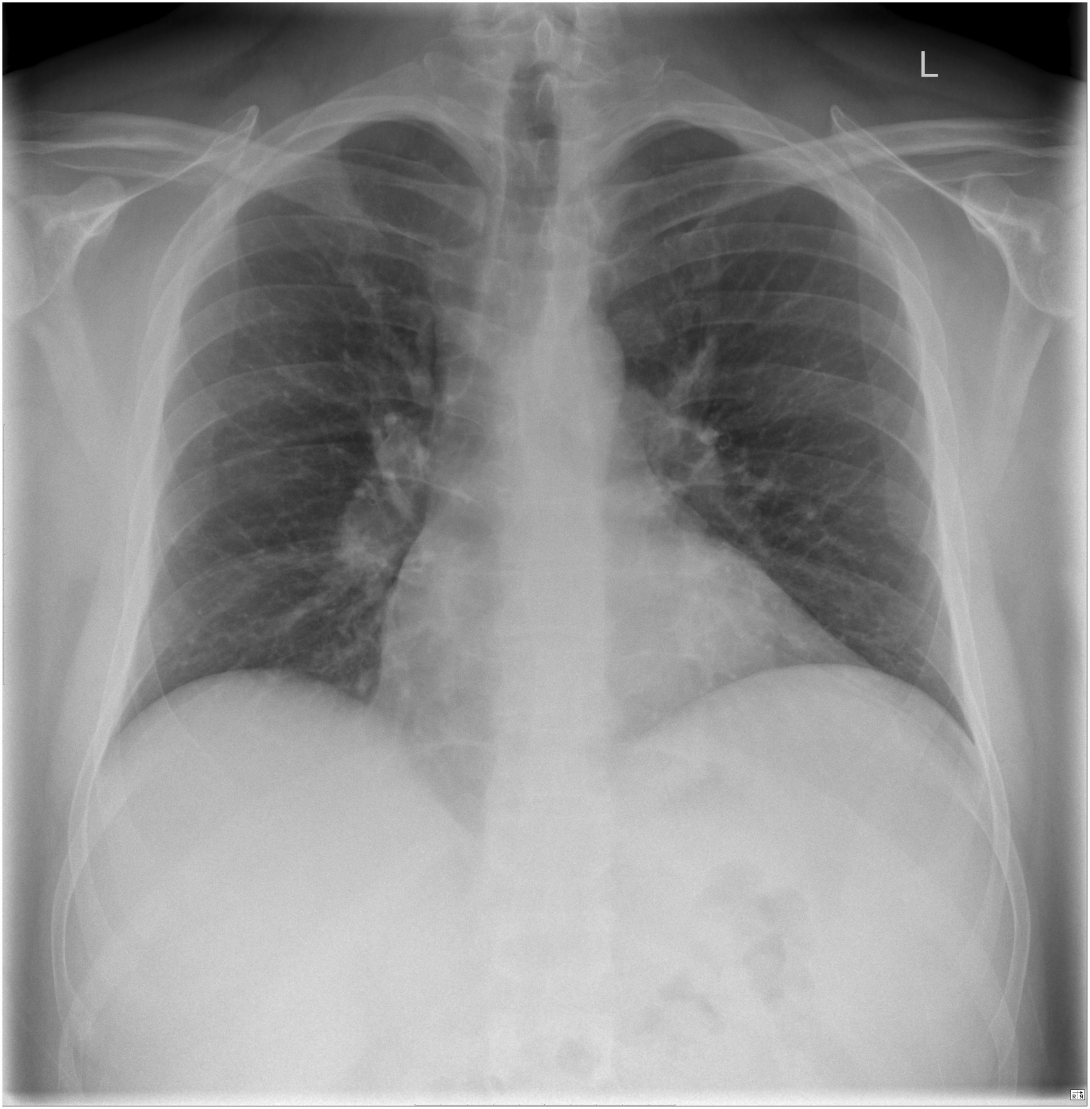
Initial chest x‐ray. Initial chest x‐ray shows a normal cardiomediastinal silhouette and no signs of infiltrates, effusion, or pneumothorax.

After the second episode of COVID‐19, the patient continued to experience body aches, headaches, brain fog, difficulty concentrating, insomnia, fatigue, dizziness, tinnitus, short‐term memory issues, and intermittent subjective fevers with exertional malaise that was severe at times, preventing him from working or performing activities of daily living. Subsequent laboratory studies showed elevation in inflammatory markers, including an erythrocyte sedimentation rate of 23 mm/h (reference range: 0–15 mm/h) and a C‐reactive protein level of 3.1 mg/dL (reference range: ≤0.5 mg/dL). However, other studies, including a complete blood count, electrolytes, blood cultures, creatinine kinase, acute hepatitis B and C antibody panels, urinalysis, were all negative. Angiotensin‐converting enzyme (ACE) levels were normal (49 U/L, reference range: 9–67 U/L). Chest x‐ray showed no abnormalities **(**Figure 1 **)**.

## 3 DIFFERENTIAL DIAGNOSIS, INVESTIGATIONS, AND TREATMENT

He was referred to sleep medicine and was found to have mild sleep apnea, for which conservative management with weight loss was recommended. He was also evaluated by a neurologist, who recommended magnetic resonance imaging (MRI) of the brain, auditory canal, and whole spine, which showed normal findings. Given the negative workup, he was diagnosed with long COVID and started on physical therapy, modafinil, and bupropion. In August 2022, he experienced a third episode of mild COVID‐19 for which he received treatment with nirmatrelvir/ritonavir (Paxlovid).

**FIGURE 2:**
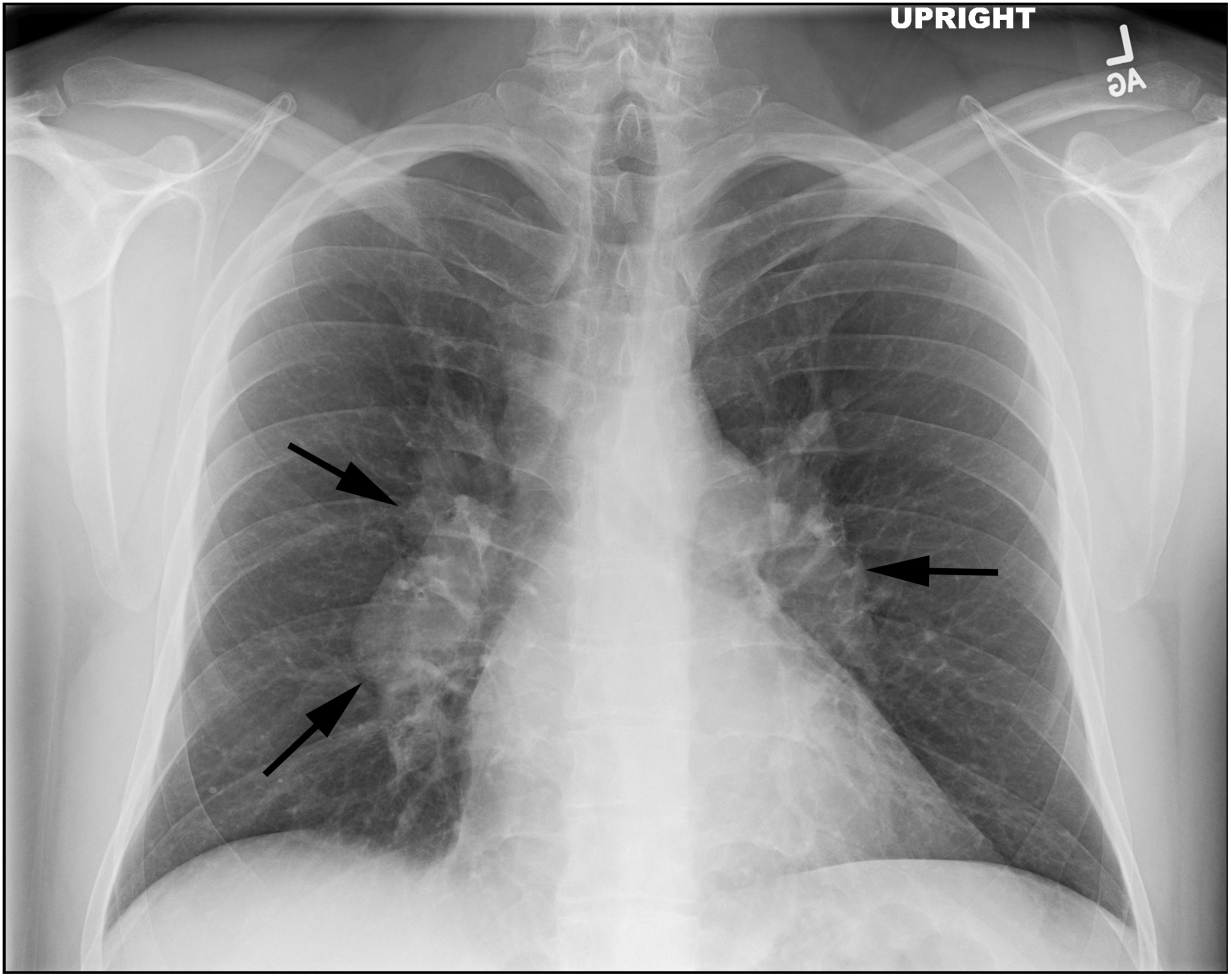
Follow‐up chest x‐ray. Follow‐up chest x‐ray reveals bilateral hilar enlargement, indicative of lymphadenopathy.

**FIGURE 3:**
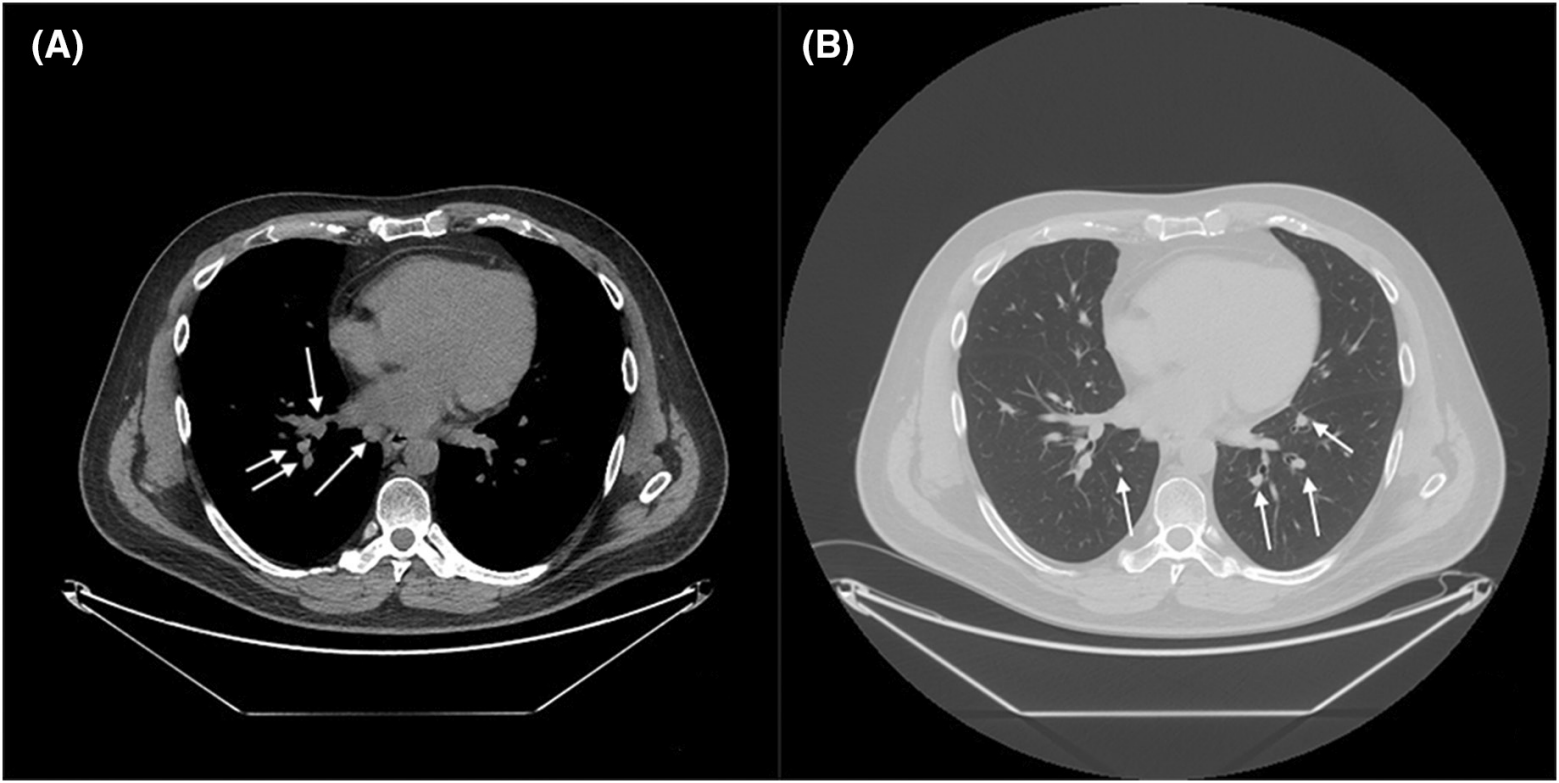
Computed tomography of the chest without contrast. Computed tomography of the chest reveals diffuse mediastinal and bilateral hilar adenopathy (A), with bulky hilar nodes measuring up to 2.7 cm on the right and 1.7 cm on the left. The largest mediastinal nodes are 1.7 cm in the right paratracheal area and 2.2 cm in the prevascular region. Additionally, pleural nodules measuring 3 mm are noted bilaterally, accompanied by scattered small bilateral lung nodules up to 5 mm, including probable intrapulmonary lymph nodes (B).

**FIGURE 4:**
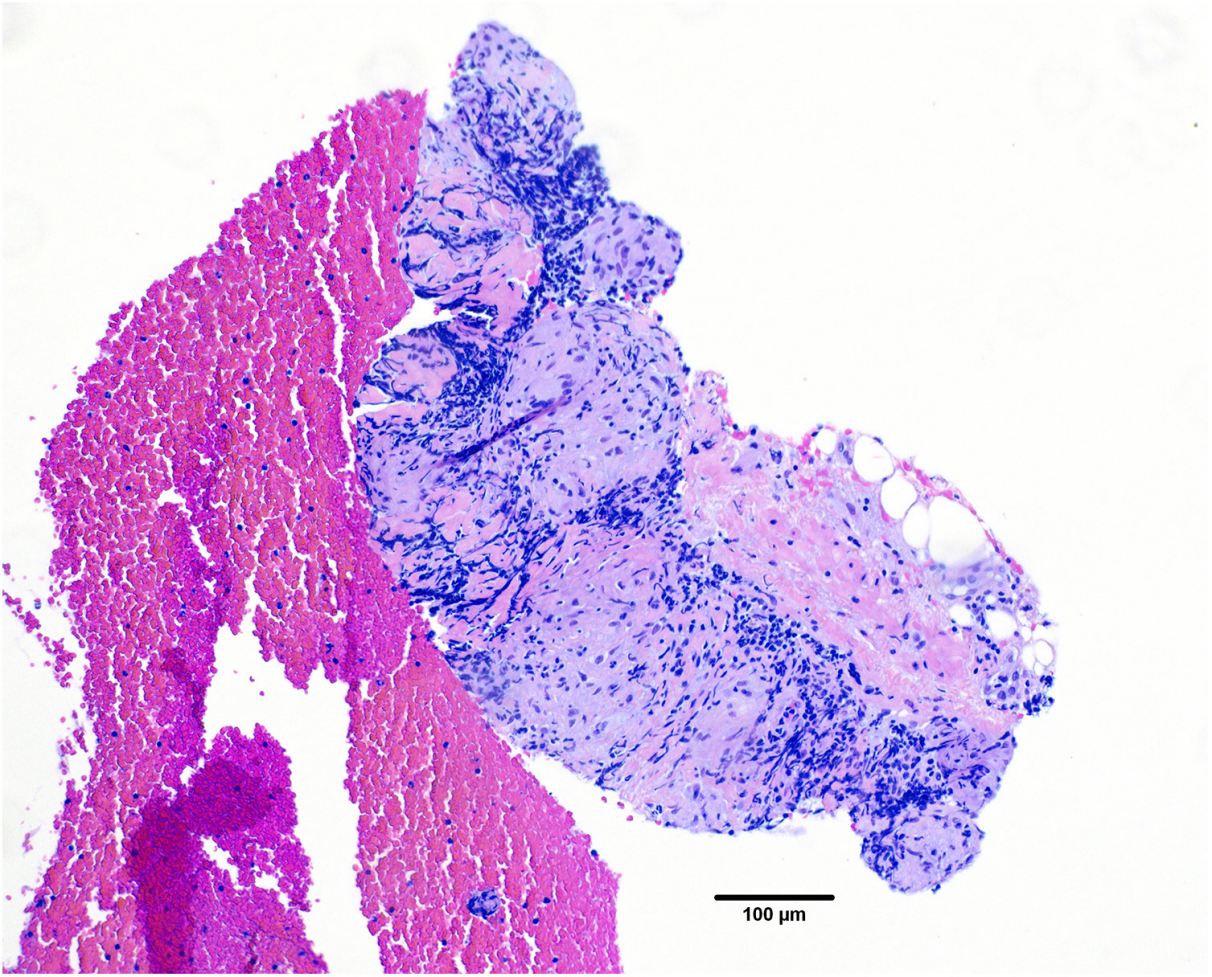
Lymph node biopsy. Station seven lymph node biopsy. Photomicrograph of lymph node tissue displaying lymphocytes, epithelioid histiocytes, and multinucleated giant cells consistent with a non‐necrotizing granuloma typical of sarcoidosis. Hematoxylin and eosin, bar = 100 μm.

The patient's persistent symptoms led to his referral to the Stanford Post‐Acute COVID‐19 Syndrome clinic in November 2022. During evaluation, a chest x‐ray was performed, revealing bilateral hilar enlargement that raised suspicion of lymphadenopathy (Figure 2). Subsequent computed tomography (CT) of the chest confirmed diffuse mediastinal and bilateral hilar bulky adenopathy (Figure 3A), accompanied by scattered pulmonary nodules bilaterally (Figure 3B). Laboratory studies indicated elevated serum calcium levels of 10.5 mg/dL (reference range: 8.6–10.3 mg/dL), with a corrected calcium level of 9.9 mg/dL, adjusted for an albumin level of 4.8 g/dL. Furthermore, the patient exhibited low parathyroid hormone levels (10 pg/mL, reference range: 16–77 pg/mL) and elevated ACE levels (114 U/L). In December 2022, the patient received a clinical diagnosis of pulmonary sarcoidosis following a transbronchial fine needle aspiration lymph node biopsy that showed non‐caseating granulomas (Figure 4).

## 4 OUTCOME AND FOLLOW‐UP

Treatment was initiated with a prednisone taper, starting at 40 mg for 2 weeks, followed by 30 mg for 2 weeks, 20 mg for 2 weeks, 10 mg for 2 weeks, and then 10 mg for 4 weeks, resulting in an improvement of almost all long COVID symptoms and normalization of ACE levels (61 U/L) after several weeks of follow‐up.

## 5 DISCUSSION

We report a case of a previously healthy White male who developed PASC following to an acute COVID‐19 episode in December 2020. Initially, his chest imaging and ACE levels were normal. However, as time progressed, the patient's symptoms persisted, worsened, and were eventually associated with mediastinal lymphadenopathy and elevated plasma ACE levels. An endobronchial ultrasound‐guided transbronchial needle aspiration later revealed non‐caseating granulomas, indicative of sarcoidosis. After a regimen of systemic steroids, the patient's long COVID symptoms completely resolved, and his ACE levels returned to normal.

The pathophysiological mechanisms underlying long COVID remain elusive. Many studies have proposed correlations with systemic inflammation, immune dysregulation, autoimmunity, and aberrant cytokine regulation.^27^, ^28^, ^29^ Other research suggests that long COVID may be linked to persistent reservoirs of SARS‐CoV‐2 in tissues and the reactivation of viruses like Epstein–Barr virus (EBV) and human herpesvirus‐6.^30^ There are also theories that implicate altered microbiome, endocrine dysregulation, microvascular blood clotting with endothelial dysfunction, and dysfunctional signaling in the brainstem or vagus nerve.^29^, ^30^, ^31^, ^32^, ^33^, ^34^ Additionally, long COVID has been associated with a host of conditions, including cardiovascular, thrombotic, and cerebrovascular diseases, type 2 diabetes, myalgic encephalomyelitis/chronic fatigue syndrome, dysautonomia, and autoimmune disorders.^29^, ^30^ These conditions may share a similar pathogenesis with long COVID.^30^ Notably, autoimmune phenomena consistently emerge as significant in theories explaining long COVID. Building on this, some population studies have observed a link between SARS‐CoV‐2 infection and a heightened risk of new‐onset autoimmune diseases post the acute infection phase (Table 1).^35^, ^36^, ^37^, ^38^ Predominantly, these affected patients were around 40 years old, female, and of White ethnicity.^35^, ^36^, ^37^ One such study highlighted that patients with a prior COVID‐19 diagnosis had a 42.63% increased likelihood of developing autoimmunity.^37^

**TABLE 1:** List of autoimmune disorders associated with SARS‐CoV‐2 infection.

Alopecia areata
Ankylosing spondylitis
Arteritis temporalis
Autoimmune hemolytic anemia
Autoimmune hepatitis
Autoimmune thyroiditis
Behcet's disease
Bullous pemphigoid
Celiac disease
Connective tissue disease
Crohn's disease
Cutaneous lupus erythematosus
Dermatomyositis
Diabetes type 1
Graves' disease
Guillain‐Barré syndrome
Hashimoto thyroiditis
Immune thrombocytopenic purpura
Multiple sclerosis
Myasthenia gravis
Polymyalgia rheumatica
Primary biliary cirrhosis
Psoriasis
Rheumatoid arthritis
Sarcoidosis
Sjögren syndrome
Systemic lupus erythematosus
Systemic sclerosis
Ulcerative colitis
Vitiligo
Wegener's disease

Over the course of decades, the scientific community has been deeply engaged in examining the association between viral infections and the initiation of autoimmune conditions, such as type 1 diabetes, multiple sclerosis, and sarcoidosis.^39^, ^40^, ^41^, ^42^, ^43^ Although some viral infections have well‐documented autoimmune effects, the links between other common viruses and autoimmune disorders are difficult to prove.^42^ Pandemics offer a unique opportunity to understand this link and the underlying pathogenesis, primarily due to their large sample sizes and the heightened vigilance of the medical community for rare outcomes.^43^

Sarcoidosis is an idiopathic granulomatous multisystem disorder characterized by dense epithelioid non‐necrotizing lesions, predominantly affecting the lungs and lymph nodes, accompanied by varying degrees of lymphocytic inflammation.^25^, ^44^ It affects all races, ethnicities, and genders with a higher prevalence among females and typically emerging in young to middle‐aged adults, peaking around 30–50 years in men and 50–60 years in women.^45^ The later diagnosis age in women compared to men may relate to menopause impacting lung function and the premenopausal protective effect of estrogen against sarcoidosis.^46^ While the exact cause remains elusive, specific environmental and occupational exposures have been linked to elevated disease rates. These include exposure to metals and silica, workers exposed to debris on September 11, 2001, firefighters, and certain infections.^26^, ^47^, ^48^ Infections with many organisms have been correlated with sarcoidosis, including *Cutibacterium acnes*, mycobacteria, and different viruses such as human herpesvirus‐8 (HHV‐8), EBV, and hepatitis C.^26^, ^49^, ^50^

So far, 15 cases, including ours, have been reported with patient‐level data linking sarcoidosis to COVID‐19 (Table 2).^51^ Out of these, 10 were diagnosed a month or longer after their initial COVID‐19 diagnosis. While some of these cases experienced symptoms associated with long COVID, such as fatigue, cough, and malaise, only our case had a PASC score of 12, aligning with the symptom‐based PASC definition proposed by Thaweethai et al.^1^ Among these cases, six developed stage ≥2 sarcoidosis,^15^, ^16^, ^17^, ^18^, ^24^ with one showing hepatic involvement,^14^ and another receiving a sarcoidosis diagnosis during the acute COVID‐19 episode.^23^ Furthermore, six presented with stage 1 sarcoidosis,^16^, ^19^, ^20^, ^21^, ^22^, ^23^ with two experiencing neurosarcoidosis,^21^, ^22^ and four displaying cutaneous manifestations, including two cases of erythema nodosum.^13^, ^14^, ^16^, ^19^ The median age of these patients was 49 years (IQR 35–54 years), with 53% of the patients being male. The reporting of race was infrequent, predominantly involving White individuals (13%). Mediastinal lymph node enlargement on imaging was observed in 60% of cases, while non‐necrotizing or non‐caseating granulomas on biopsy were present in 87%. Elevated ACE levels were documented in only four cases. Additionally, 60% of the cases received systemic steroids for treatment.^13^, ^14^, ^15^, ^16^, ^17^, ^18^, ^19^, ^20^, ^21^, ^22^, ^23^, ^24^

**TABLE 2:** Reported cases of sarcoidosis following COVID‐19.

Case	Age	Sex	Race	Comorbidities	Time from COVID‐19 to diagnosis	PASC score^1^	Sarcoidosis diagnosis	Chest x‐ray scoring system (Prnjavorac et al.)	Other changes	Treatment	Reference
1	72	Male	White	Asthma, hypertension, hyperlipidemia, obstructive sleep apnea, seizure disorder	14 days	NA	Painful, violaceous nodules on the anterior shins, lateral thighs, submental neck, and glabella. Biopsies taken from the leg and submental neck painful, violaceous nodules with well‐formed non‐caseating granulomas	NA	None	Clobetasol ointment	Behbahani et al.^13^
2	55	Female	NA	Hypertension, hyperlipidemia, hypothyroidism	2 months	0	Swelling of old scars, papules at the sites of botulinium toxin‐A, and subcutaneous nodules. Normal chest x‐ray. Excisional biopsy from an infiltrated plaque with non‐caseating granulomas	NA	NA	None	Polat Ekinci et al.^14^
3	50	Male	NA	Familial hypercholesterolemia, fatty liver disease, type 2 diabetes	9 months	2	Developed pleurisy. CT chest with mediastinal lymphadenopathy and scattered nodules. PET with uptake concerning for reactive nodes, chronic granulomatous disease or lymphoproliferative disease. Lymph node aspirate with non‐caseating granulomas and liver biopsy with granulomatous inflammation	NA	ALT 104, AST 81, Alkaline Phosphatase 216, Total Bilirubin 1.1. Liver ultrasound and transient elastography with steatosis and infiltrating liver disease	Prednisone 40 mg daily, followed by azathioprine and budesonide	Cioffi et al.^15^
4	32	Female	NA	NA	14 days	NA	Developed tachycardia. Clinical examination with erythema nodosum lesions of the legs and inflammatory arthralgia. CT chest with mediastinal and hilar lymphadenopathy	NA	NA	NA	Mertz et al.^16^
5	51	Female	NA	Familial history of sarcoidosis	1 month	0	Developed painful latero‐cervical lymphadenopathies. PET‐CT with supra and infra‐diaphragmatic hypermetabolic lymph nodes. Lymph node biopsy with non‐caseating granulomas	NA	NA	NA	Mertz et al.^16^
6	32	Female	NA	NA	1 month	0	Isolated rythema nodosum in lower extremities	NA	NA	NA	Mertz et al.^16^
7	NA	NA	NA	None	8 months	0	Developed persistent dyspnea. PET‐CT with uptake in lung parenchyma and bilateral hilar mediastinal uptake. Lymph node biopsy with non‐necrotizing sarcoid type granulomatous inflammation	NA	NA	High‐dose corticoids	Rodríguez‐Alfonso et al.^17^
8	61	Male	NA	Prostate cancer, chronic kidney disease	14 months	4	Developed persistent respiratory symptoms (not described), fatigue, and weight loss. CT chest with pulmonary nodules and diffuse reticulation. FDG‐PE with extensive bilateral high level FDG‐avidity of mediastinal and hilar lymph nodes. ACE of 207 U/L. Lung nodule core biopsy with non‐necrotizing, well‐formed sarcoid‐like granulomas	7	ESR of 25 mm/h, hypercalcemia of 10.6 mg/dL	Prednisone 20 mg tapered to 10 mg daily	Capaccione et al.^18^
9	31	Male	NA	NA	6 months	0	Widespread, itchy, and extensive erythematous lesions. CT chest with mediastinal lymphadenopathy. ACE of 145 U/L. Skin punch biopsy with compact non‐caseating epithelioid cell granulomas	NA	None	Prednisolone 40 mg daily	Rabufetti et al.^19^
10	38	Male	NA	None	14 days	NA	CT chest with mediastinal and hilar lymphadenopathy. ACE <40 micrograms/L. Fine needle aspirationbiopsy with non‐necrotizing granulomatosis	NA	AST of 87 U/L, CRP of 63 mg/dL, D‐dimer 1.28 ug/mL	None	Kucukardali et al.^20^
11	48	Female	NA	NA	1 month	0	Developed a reactive arthritis, left facial lower motor neuron weakness, cranial neuropathies, and bilateral leg weakness. FDG‐PET showed mediastinal and axillary hilar lymphadenopathy. ACE reported elevated at 2.07umol/min/L. Neck lymph node biopsy with non‐necrotizing granulomatous inflammation	NA	MRI brain with bilateral enhancement of trigeminal and facial nerves	High dose steroids	Robinson et al.^21^
12	51	Female	NA	NA	NA	NA	Multiple cranial neuropathies and paresthesia. Abnormal CT chest. Lymph node biopsy with non‐caseating granulomas	NA	CSF with mild protein elevation and elevated CD4:CD8 ratio. Elevated serum sIL‐2R	Steroids, followed by methotrexate	Mafla Delgado et al.^22^
13	35	Male	Asian	None	10 weeks	11	Developed low‐grade fever, malaise, cough and post exertional dyspnea. Chest x‐rays with enlarged bilateral hilar area. Chest CT with bilateral hilar, paratracheal, and subcarinal lymphadenopathy. ACE 20.3 U/L Transbronchial needle aspiration of mediastinal lymph nodes showed well‐formed non‐necrotizing granulomas with epithelioid histiocytes and lymphocytes	4	Panuveitis, papillitis and retinal vasculitis in both eyes	Prednisolone 60 mg daily tapered to 20 mg over 1 month and maintained at this dose	Somboonviboon.^22^, ^23^
14	64	Male	NA	COPD, atrial fibrillation, heart failure, type 2 diabetes, hypertension, stroke, thyroid disease, smoking history	0	NA	Acute COVID‐19 with CT chest showing small bilateral pleural effusions a lobulated mass in the right upper lobe with mediastinal lymphadenopathy. ACE <25 IU/L. CT‐guided biopsy and lymph node aspiration with chronic inflammation and vague epitheloid non‐caseating granulomas	0	None	Methylprednisolone, doxycycline, ceftriaxone, followed with prednisolone 40 mg daily subsequently tapered to 4 mg daily	Pokhriyal.^24^
15	35	Male	White	None	24 months	12	Post‐acute sequelae of COVID‐19. Chest x‐ray and CT chest with diffuse mediastinal and bilateral hilar bulky adenopathy. ACE 114 U/L. Lymph node biopsy with non‐caseating granulomas	4	Elevated calcium levels, C‐reactive protein, and erythrocyte sedimentation rate	Prednisone taper	Rodriguez‐Nava et al.

Abbreviations: ACE, angiotensin‐converting enzyme; ALT, alanine transaminase; AST, aspartate aminotransferase; COVID‐19; coronavirus disease 2019; CSF, cerebrospinal fluid; CT, computed tomography; FDG, fludeoxyglucose F18; NA, not applicable or not available; PASC, post‐acute sequelae of SARS‐CoV‐2 infection; PET, positron emission tomography.

The reported cases and a large cohort study provides compelling evidence to support an association between SARS‐CoV‐2 and sarcoidosis.^13^, ^14^, ^15^, ^16^, ^17^, ^18^, ^19^, ^20^, ^21^, ^22^, ^23^, ^24^, ^37^ Certainly, there are comparable immune response pathways in both sarcoidosis and convalescent COVID‐19 patients that contribute to granuloma formation. These include disruption of the renin–angiotensin system, elevated CD4/CD8 ratio in bronchoalveolar lavage fluid, accumulation of multinucleated giant cells in lung tissue, polarization of Th17 cells into Th1 cells, increased production of type II interferon (IFN‐γ), dysregulated autophagy, upregulation of cytokines, and reduced PD‐1 expression.^43^, ^48^ Furthermore, clinical manifestations such as mild fever, fatigue, joint pain, cognitive disorders, and weight loss exhibit overlapping characteristics among patients with both sarcoidosis and long COVID.^48^

In conclusion, long COVID is a multifaceted condition that persists in certain individuals following a SARS‐CoV‐2 infection, with symptoms extending beyond the acute phase and potentially giving rise to various new‐onset conditions. The reported case highlights the intricate interplay between long COVID and sarcoidosis, shedding light on their overlapping clinical features and immunological pathways. The emergence of SARS‐CoV‐2 has brought to the forefront new evidence suggesting a connection between infections and sarcoidosis. When managing patients with long COVID, healthcare providers should prioritize assessing for autoimmune conditions, as this approach could potentially offer tailored treatment options.

## AUTHOR CONTRIBUTIONS

**Guillermo Rodriguez‐Nava:** Visualization; writing – original draft; writing – review and editing. **Vanessa El Kamari:** Writing – review and editing. **Harvey Chang:** Writing – review and editing. **Goar Egoryan:** Writing – review and editing. **Hector F. Bonilla:** Supervision; visualization; writing – review and editing.

## FUNDING INFORMATION

This research did not receive any specific grant from funding agencies in the public, commercial, or not‐for‐profit sectors.

## CONFLICT OF INTEREST STATEMENT

The authors declare that they have no competing interests.

## CONSENT

Written informed consent was obtained from the patient to publish this report in accordance with the journal's patient consent policy.

## Data Availability

Data sharing is not applicable to this article as no datasets were generated or analyzed during the current study.
